# Turkish Validity and Reliability Study of Jessa Atrial Fibrillation Knowledge Questionnaire (JAKQ)

**DOI:** 10.5152/eurasianjmed.2021.20247

**Published:** 2021-10

**Authors:** İbrahim Özlü, Atıf Bayramoğlu

**Affiliations:** 1 Department of Emergency, Atatürk University School of Medicine, Erzurum, Turkey; 2 Department of Emergency Medicine, Atatürk University School of Medicine, Erzurum, Turkey

**Keywords:** Atrial fibrillation, Validity, Reliability

## Abstract

**Objective:**

Atrial fibrillation (AF) is the most commonly encountered arhythmia in clinical practice and constitutes one-third of the hospitalizations related to cardiac dysrhythmia. Furthermore, it is the most common reason for hospital admissions involving dysrhythmia complaints and is associated with the decrease in quality of life, functional capacity, cardiac performance, and lifespan.

**Materials and Methods:**

This methodological study, which aims to assess the validity and reliability of the JAKQ, was conducted with patients (n = 175) who were admitted to the emergency clinic of Atatürk University Research Hospital between December 2016 and June 2018 and who were diagnosed with atrial fibrillation and met the inclusion criteria. The data were collected using a “Personal Information Form” and the “Jessa Atrial Fibrillation Knowledge Questionnaire (JAKQ).

**Results:**

The exploratory factor analysis carried out to determine the construct validity of the scale showed that it had a three-dimensional structure and that its factor loads were within the appropriate range. The internal consistency analyses of the scale revealed that the Cronbach’s alpha coefficient was 0.84 and that the item-total score correlations were at an adequate level. The correlation value of the test-retest conducted to test the time invariance of the scale was found to be 0.87, and there was a statistically significant relationship between the two applications (*P* < .001).

**Conclusion:**

The study results showed that the structure of the Turkish version of the Jessa Atrial Fibrillation Questionnaire was similar to the structure of the original scale, that its validity and reliability were considerably high and that it could be used in Turkey.

## Introduction

Atrial fibrillation (AF) is a complex arrythmia which is characterized by irregular ventricular contraction induced by fast, irregular, and ineffective atrial activity.^1^ AF is the most commonly encountered arrythmia in clinical practice and constitutes one-third of the hospitalizations related to cardiac dysrhythmia.^2^ One of the most dangerous and destructive complications associated with AF is stroke. Patients with AF have a fivefold increased risk of stroke. Generally, 20% of strokes are related to AF and those related to AF tend to be more fatal and intense. Additionally, AF is a strong and independent risk factor for mortality and morbidity.^3^ Due to the high stroke rates associated with AF, early diagnosis of AF and effective anticoagulation of patients with AF are necessary to prevent AF-induced strokes. Another important issue to note is that AF-related stroke causes irreversible serious organic damages.^4^

Considering the seriousness of AF, patients need to be informed about the condition as soon as they are diagnosed with it in order for them to become more competent in AF management. The primary training subjects taught to these patients should include general information about AF, the effects of the medications, medication-related complications, thromboembolic complications related to AF, and emergency symptoms.^5^ This training should be provided within the framework of a program, for which the most important data constituting it is the patients’ level of knowledge about their illness. However, there are numerous studies showing that patients have a critically low level of information about arrhythmia and oral anticoagulant therapy (OAC).^6^ The international research on this common problem and a paper published by the European Heart Rhythm Association (EHRA) point out that patient training is an important issue in the care of AF.^7,8^ Understanding this, specific measurement tools that are capable of assessing the level of knowledge of AF patients and that have confirmed validity and reliability are required. Given that no such measurement tool was found in Turkey, this study aims to assess the Turkish validity and reliability of the Jessa Atrial Fibrillation Knowledge Questionnaire (JAKQ).

## Materials and Methods

### Type of Study

This study was conducted as a methodological study to assess the validity and reliability of the Turkish adaptation of the “Jessa Atrial Fibrillation Knowledge Questionnaire” for use in Turkish society.

### Time and Place

This study was conducted in the emergency medical clinic of Atatürk University Research Hospital between December 9, 2016 and June 25, 2018.

### Population and Sample

The study population was composed of patients who were admitted to the Emergency Medical Clinic of the Atatürk University Research Hospital between December 9, 2016 and June 25, 2018 and who were diagnosed with Atrial Fibrillation; the study sample consisted of those patients who met the inclusion criteria (n = 175).

For the adaptation of a scale to another culture, the sample size is required to be at least five to ten times greater than the scale’s number of items. Since there were 16 items on the scale, it was determined that the sample size should be between at least 80-160. The actual sample size in this study met this requirement, being 175.

### Data Collection

The data were collected through face-to-face interviews using the Personal Information Form and the Jessa Atrial Fibrillation Knowledge Questionnaire (JAKQ) following completion of the necessary procedures on the patients and stabilization of their condition. The individuals who participated in the study were informed about the study and their verbal consents to participate were obtained. During data collection, the retest method was used to determine the reliability of the JAKQ. The scale was reapplied to the participating patients through phone calls 1 month later. It took approximately 8 to 10 min to fill out the Personal Information Form and the scale.

## Data Collection Tools

### Personal Information Form

This form was developed by the researchers in compliance with the literature. It consists of questions about patients’ descriptive and illness-related characteristics.

### Jessa Atrial Fibrillation Knowledge Questionnaire (JAKQ)

The JAKQ was developed by Desteghe et al^6^. in 2016 to assess the level of knowledge AF patients have about their illness.^6^ The specific scale is an easy-to-use, short, complete, and valid measurement tool, which can also be used for personal patient training.

The scale includes 16 items, with 8 having questions about “general information on AF”, 5 about “oral anticoagulant treatments”, and 3 about “vitamin K antagonists (VKA)” or “new anticoagulants (non-vitamin K antagonist oral anticoagulants = NOAC)”. For every item, responses of “false” or “I do not know” were scored 0 points and while those of “correct” were scored 1 point. The total score of the scale is obtained by summing the scores from every item.

### Data Analysis

The data were evaluated using the IBM SPSS Statistics version 22 (IBM SPSS Corp.; Armonk, NY, USA) program and were coded to create a database. Demographic data derived from the personal information form were statistically analyzed. The validity of the questionnaire was determined through the opinions of specialists, the Barlett Test, the Kaiser-Meyer-Olkin Index (KMO), the Exploratory Factor Analysis, the Confirmatory Factor Analysis, the Principal Component Analysis, and the Varimax Rotation Test. As part of the reliability analysis, the Cronbach’s Alpha Coefficient and Pearson’s Product-Moment Correlation Coefficient were used to determine internal consistency and homogeneity.

### Ethical Considerations

Ethical approval for the study was obtained from the Ethical Committee of Erzurum Atatürk University Medical Faculty (08.12.2016, 7/34). The participants were informed about the objective and method of the study and the time needed to be allocated for the study. Moreover, they were told that participation in the study would pose no risks and was voluntary and that they could withdraw from the study at any time.

The author of the original scale gave permission for the Turkish adaptation of the JAKQ, which was developed by Desteghe et al.^6^ in 2016.

## Results

In examining the introductory characteristics of the patients, it was found that 56% were female, 91.4% were married, 60% had an extended family structure, 54.3% were housewives, 73.7% had a moderate level income, and 71.4% resided in the city. The mean age of the participants was found to be 71.69 ± 10.15, and the mean number of children was 4.88 ± 2.27.

Among the patients, 34.9% were admitted with breathing difficulties, 32.6% were diagnosed with long-standing persistent AF, 32.6% had been diagnosed with AF for 1-5 years, 28.6% used APT as anticoagulation/ antithrombotic treatment, 58.3% were hospitalized more than two times, and 78.9% were diagnosed with a chronic illness. Patients’ mean CHA_2_DS_2_VAS_c_ score was found to be 3.73 ± 1.78 and their mean HAS-BLED bleeding score was 2.50 ± 1.45.

Kaiser-Meyer-Olkin (KMO) and Bartlett’s tests were conducted to determine whether the sample size was sufficient, and the data were suitable for the factor analysis before the principal component analysis, which was conducted to provide more precise results in the study.

Examination of the Kaiser-Meyer-Olkin and Bartlett’s test results (Table 1) showed that the KMO coefficient for the General Information about Atrial Fibrillation subscale was 0.800, 0.765 for the Anticoagulant Medications subscale, and 0.702 for the Vitamin K Antagonists and New Anticoagulant Medication subscale. These results indicate that the sample size was suitable for the factor analysis. Similarly, the chi-square values of the Bartlett’s test were significant at the *P* < .001 significance level and were suitable for the factor analysis.

Table 2 **s**hows the scale’s item-total correlations and Cronbach’s alpha coefficients. The mean Cronbach’s alpha coefficient of the General Information about Atrial Fibrillation 0.783; mean Cronbach’s alpha coefficient of the Anticoagulant Medications subscale 0.771; and finally, the mean Cronbach’s alpha coefficient of the Vitamin K Antagonists and New Anticoagulant Medications subscale 0.615. The Cronbach’s alpha total value was found to be 0.844.

As in the original scale, the data were evaluated by direct oblimum rotation. Further analysis showed that the scale was also suitable for the three-subscale structure in the Turkish language, as was the case in its original language. The factor loads of the General Information about Atrial Fibrillation subscale ranged from 0.517 to 0.808, and the factor loads of all items were over 0.40. The explained variance was 41.110%.

**Figure 1.**
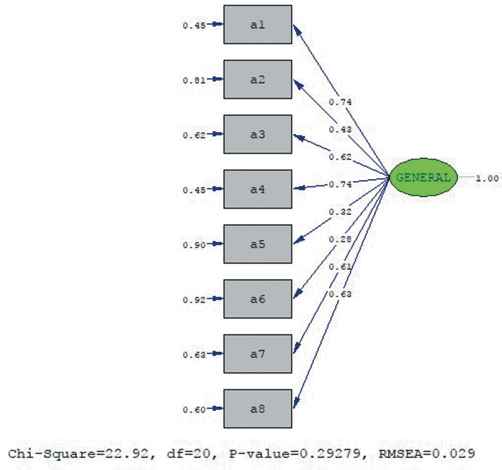
The PATH diagram for the Turkish version of the general information about atrial fibrillation subscale

Figure 1 shows the PATH diagram for the factor loads of the items in the General Information about Atrial Fibrillation subscale. Figure 1 reveals that the scale was accepted with its original structure without applying any modifications.

**Figure 2.**
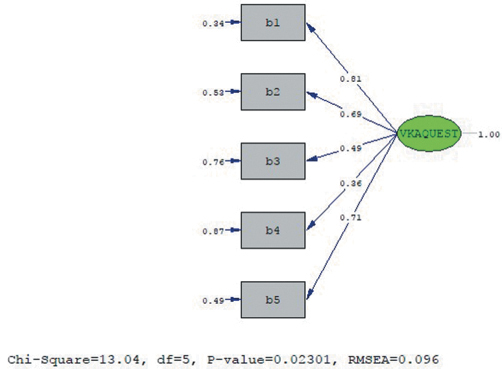
PATH diagram of the Turkish version’s anticoagulant medications subscale

Figure 2 shows the Path diagram for the factor loads of the items in the Anticoagulant Medications subscale. Figure 2 shows that the scale was accepted with its original structure without applying any modifications.

**Figure 3.**
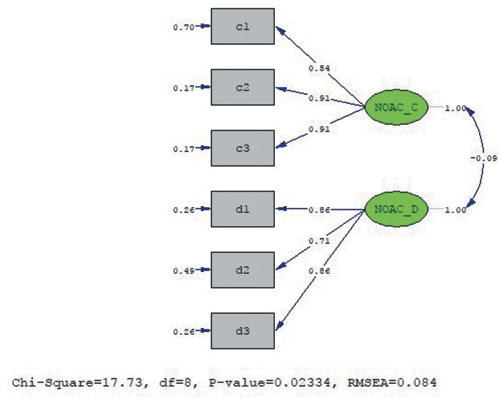
PATH diagram for the Turkish version of the vitamin K antagonists and new anticoagulant medications subscale

Figure 3 shows the Path diagram for the factor loads of the items in the Vitamin K Antagonists and New Anticoagulant Medications subscale. Figure 3 shows that the scale was accepted with its original structure without applying any modifications.

Confirmatory Factor Analysis (CFA) was performed to test the suitability of the three-factor structure revealed through the explanatory factor analysis. Table 3 shows the fit index values, and normal and acceptable values for JAKQ. The related fit index values indicate that this version of the scale was acceptable, and that it was composed of three subscales. The CFA performed for the original scale as part of the explanatory factor analysis also showed that the scale was composed of three subscales.

To determine the time invariance of the scale, test-retest was carried out and the Pearson Moments Multiplication results were examined. Table 4 shows that the correlation value of the relationship between the first and second application scores of the study was significant at the *P* < .000 significance level, indicating that the results of the first and second measurements of the scale, which were made at one-month intervals, were similar.

## Discussion

No specific scale was found in Turkey that assesses the knowledge level of AF patients. Thus, this study performed a Turkish validity and reliability study of the Jessa Atrial Fibrillation Knowledge Questionnaire. In this section, the language validity, content validity, construct validity, internal consistency, and findings related to certain variables tested as part of the validity and reliability study of the 16-item, 3-factor scale are discussed.

### Discussion on Results of the Language Validity

The first step in scale adaptation studies is the translation of the original scale to the target culture’s language.^9,10^

The translation-retranslation method, which is the most commonly used method thro-ughout the world, was used for the language adaptation of the scale to minimize the conceptualization and expression differences.^10,11^

The results from this process performed in this study showed that the Turkish version of the JAKQ is an appropriate measurement tool in terms of language validity.

### Discussion on Results of the Content Validity

The Davis technique is used to assess content validity and involves obtaining the opinion of experts on the subject.^12^

For the content validity, the scale was presented to ten expert academicians, who were asked to evaluate the measurement level of every item, after the translation process. The literature on this subject states that the number of experts should be between 3 and 20 for content validity calculations.^11^ This study asked the opinion of ten experts, a number that falls well within the recommended range.

It was further stated in the literature that in content validity calculations made with the Davis technique, the CVI score should be 0.80 or above.^13^ This study determined that the CVC/CVI scores of all scale items were over 0.80. Considering these results, JAKQ was found to be an appropriate measurement tool in terms of content validity.

### Discussion on Results of the Construct Validity

The KMO value is expected to be over 60 for the factor analysis to be at a good level.^12^ In this study, the KMO value was found to be 0.79, meaning that the sample size was adequate for the factor analysis.^13^ In the original scale, the KMO values were found to be 0.774 for the General Information about Atrial Fibrillation subscale, 0.668 for the Anticoagulant Medications subscale, and 0.670 for the Vitamin K Antagonists and New Anticoagulant Medications subscale.^6^ In the Turkish adaptation study of the JAKQ, the KMO values were found to be 0.800 for the General Information about Atrial Fibrillation subscale, 0.765 for the Anticoagulant Medications subscale, and 0.702 for the part Vitamin K Antagonists and New Anticoagulant Medications subscale. These results showed that the sample size was adequate for the factor analysis.

In scale adaptations, significant Bartlett’s test results indicate a good level of sample size and a suitable correlation matrix for factor analysis.^12,13^ The Bartlett’s test value of the original scale was reported to be *P* = .000,^6^ which was the same for this study, which means that the data were normally distributed, measurement results were not affected by the sample size, and the sample size was adequate and suitable for factor analysis.

Exploratory and confirmatory factor analyses were used to determine the construct validity of the JAKQ. Furthermore, as part of the exploratory factor analysis, factor loads matrix of the items was examined to determine the relationship between the items and factors and the number of subscales. In this analysis, the data were assessed by direct oblimum rotation, as in the original scale.^12,14,15^ As a result of the analysis, it was found that the scale factor structure is suitable for the three-subscale structure. The literature recommends that factor loads be 0.30 and above.^12^ In this study, the factor loads of every item were over 0.40 and ranged from 0.51 and 0.91, as was the case for the original scale.^6^ These results indicate that the factor load of the scale items was high.

An explained variance between 40% and 60% indicates that the factor structure is adequate.^16,17^ In this study, the explained variance was found to be 41.11% for the General Information about Atrial Fibrillation subscale, 44.05% for the Anticoagulant Medications subscale, and 75.66% for the Vitamin K Antagonists and New Anticoagulant Medications subscale. The factor analysis results show that the item factor loads and explained variance were at an adequate level. After the exploratory factor analysis, the suitability of the data set to the theoretical structure was investigated by analyzing the scale items with Confirmatory Factor Analysis (CFA).^18^

The three-factor structure of the 16-item JAKQ is appropriate and corresponds to the scale’s construct validity.

### Discussion on the Results of the Internal Consistency

The Cronbach’s alpha coefficient is between 0 and 1,^14,19^ and the higher the Cronbach’s alpha coefficient of a scale, the more the scale includes consistent items that measure the elements of the same property.^19^ In this study, the total Cronbach’s alpha coefficient of the JAKQ was found to be 0.84, while for the General Information about Atrial Fibrillation subscale it was 0.78, for the Anticoagulant Medications subscale it was 0.77, and for the Vitamin K Antagonists and New Anticoagulant Medications subscale it was 0.61. In the original scale, the Cronbach’s alpha coefficient for the General Information about Atrial Fibrillation subscale was 0.67, for the Anticoagulant Medications subscale it was 0.60, and for the Vitamin K Antagonists and New Anticoagulant Medications subscale it was 0.52.^6^ In the literature, it is stated that a Cronbach’s alpha coefficient of between 0.60 and 0.80 is sufficient for the scale to be used in a study.^19^ The Cronbach’s alpha coefficients of this study, therefore, are at a sufficient level.

A higher item-total correlation indicates higher effectivity of that item, while a lower correlation coefficient indicates that a scale item is not reliable enough.^14,19^ For an item to be considered acceptable, the item-total correlation coefficient should be positive and at least 0.20. Items that have an item-total correlation of below 0.20 should be excluded from the scale, as they would affect the reliability of the scale in a negative way.^10^ It was found in this study that the total score correlation of all items was between 0.35 and 0.60, which is considered a sufficient level. The results, therefore, confirm that the JAKQ is a reliable tool for assessing the knowledge level of AF patients.

## Discussion of the Results on the Time Invariance (Test-Retest)

In the time invariance criterion, the results from similar repetitive measurements of the scale performed at different times are evaluated.^11^ A positive higher-level significant relationship was found between those two measurements. Desteghe et al^6^. used the test-retest reliability method in their study and found that there was a positive significant relationship between two measurements,^6^ meaning that the results obtained from the first and second measurement of the scale were similar.

The results from the analyses conducted to determine the reliability of the scale showed that the reliability of the JAKQ is high.

In conclusion, The scale is composed of three subscales and has a high alpha coefficient (total JAKQ α: 0.84). All of the statistical assessments showed that the scale is a valid and reliable measurement tool for the Turkish society.

**Main Points**

- The specific scale is an easy-to-use, short, complete, and valid measurement tool.
- It can also be used for personal patient training.
- Its validity and reliability were considerably high, and that it could be used in Turkey.

## Figures and Tables

**Table 1.** Kaiser-Meyer-Olkin (KMO) and Bartlett’s Test Results

Tests (n = 175)	Results	*P*
Subscale on general information about atrial fibrillation		
KMO	0.800	
Bartlett’s test	*X*^2^ = 336.676	.000
Subscale on anticoagulant medications		
KMO	0.765	
Bartlett’s test	*X*^2^ = 278.255	.000
Subscale on vitamin K antagonists and new anticoagulant medications		
KMO	0.702	
Bartlett’s test	*X*^2^ = 484.964	.000

**Table 2.** Item-Total Correlations and Cronbach’s α Coefficients

	Cronbach’s α	
General information about atrial fibrillation subscale		0.783
Anticoagulant medications subscale		0.771
Vitamin K antagonists and new anticoagulant medications subscale		0.615
Total Cronbach’s α		0.844

**Table 3.** Found Fit Indices Values and Normal and Acceptable Values

	Index	Normal value	Acceptable value	Found value
General information about atrial fibrillation subscale	*x*^2^/SD	<2	<5	1.15
	GFI	>0.95	>0.90	0.93
	AGFI	>0.95	>0.90	0.88
	CFI	>0.95	>0.90	0.99
	RMSEA	<0.05	<0.08	0.029
	SRMR	<0.05	<0.08	0.059
Anticoagulant medication subscale	*x*^2^/SD	<2	<5	2.61
	GFI	>0.95	>0.90	0.97
	AGFI	>0.95	>0.90	0.90
	CFI	>0.95	>0.90	0.97
	RMSEA	<0.05	<0.08	0,096
	SRMR	<0.05	<0.08	0.10
Vitamin K antagonists and new anticoagulant medications subscale	*x*^2^/SD	<2	<5	2.22
	GFI	>0.95	>0.90	0.99
	AGFI	>0.95	>0.90	0.99
	CFI	>0.95	>0.90	0.98
	RMSEA	<0.05	<0.08	0.084
	SRMR	<0.05	<0.08	0.017

**Table 4.** Retest Results of the JAKQ (N = 78)

Subscales		Jessa atrial fibrillation knowledge questionnaire		
		General information about atrial fibrillation	Anticoagulant medications	Vitamin K antagonists and new anticoagulant medications
General information about atrial fibrillation	*r*	0.877		
	*P*	**.000**		
Anticoagulant medications	*r*		0.870	
	*P*		**.000**	
Vitamin K antagonists and new anticoagulant medications	*r*			0.879
	*P*			**.000**
